# Trends in the Incidence and Survival Rates of Primary Ovarian Clear Cell Carcinoma Compared to Ovarian Serous Carcinoma in Korea

**DOI:** 10.3389/fonc.2022.874037

**Published:** 2022-04-07

**Authors:** Se Ik Kim, Hyeong In Ha, Kyung Jin Eoh, Jiwon Lim, Young-Joo Won, Myong Cheol Lim

**Affiliations:** ^1^ Department of Obstetrics and Gynecology, Seoul National University College of Medicine, Seoul, South Korea; ^2^ Department of Obstetrics and Gynecology, Pusan National University Yangsan Hospital, Yangsan, South Korea; ^3^ Department of Obstetrics and Gynecology, Yongin Severance Hospital, Yonsei University College of Medicine, Yongin, South Korea; ^4^ Division of Cancer Registration and Surveillance, National Cancer Center, Goyang, South Korea; ^5^ Department of Health Administration, Yonsei University, Wonju, South Korea; ^6^ Center for Gynecologic Cancer and Center for Clinical Trial, Hospital, National Cancer Center, Goyang, South Korea; ^7^ Department of Cancer Control and Policy, National Cancer Center Graduate School of Cancer Science and Policy, National Cancer Center, Goyang, South Korea; ^8^ Rare & Pediatric Cancer Branch and Immuno-oncology Branch, Division of Rare and Refractory Cancer, Research Institute, National Cancer Center, Goyang, South Korea

**Keywords:** ovarian cancer, histologic subtype, clear cell carcinoma, serous carcinoma, incidence, survival

## Abstract

**Objective:**

To compare the incidence and survival rates of primary ovarian clear cell carcinoma (OCCC) and ovarian serous carcinoma (OSC) from a nationwide collected database.

**Methods:**

We extracted information of patients with primary OCCC and OSC from the Korea Central Cancer Registry recorded between 1999 and 2018, including age at diagnosis and the Surveillance, Epidemiology, and End Results summary stage. Age-standardized incidence rates (ASRs) and annual percent changes (APCs) were calculated. Baseline characteristics and overall survival (OS) were compared between the OCCC and OSC groups.

**Results:**

Overall, the incidence rate of primary OCCC increased markedly from 1999 (ASR, 0.16/100,000) to 2018 (0.76/100,000) (APC, 7.85%; *P*<0.0001). Patients with OCCC were significantly younger and had early-stage disease more frequently than those with OSC. Patients diagnosed with OCCC before the age of 50 showed better OS than those diagnosed after the age of 50 (*P*=0.0048). The 5-year OS of the OCCC group did not differ by study period [73.5% (1999–2008) vs. 75.4% (2009–2018), *P*=0.3187], whereas the 5-year OS of the OSC group improved from 54.4% to 58% (*P*=0.0003).

**Conclusions:**

Our nationwide registry-based study demonstrated that the incidence of OCCC in Korea increased significantly from 1999 to 2018. Early-stage OCCC had a relatively good prognosis, but advanced-stage OCCC had a worse OS than advanced-stage OSC. Therefore, the development of optimal treatment strategies for OCCC is warranted.

## Introduction

Ovarian cancer, one of the deadliest female cancers, is a global burden, with an estimated 314,000 new cases and 207,000 deaths each year (1). Due to the absence of disease-specific symptoms and screening tools, most ovarian cancer cases are diagnosed at an advanced stage, resulting in high recurrence and mortality rates despite initial treatment. While the incidence rate of ovarian cancer is decreasing in the United States (2), it has progressively increased in Korea since 1999 (3), accompanied by a Western lifestyle and an increase in obese women (4). Ovarian cancer comprises various histological entities with distinct etiologies, molecular biology, and clinical features. Approximately 90% of ovarian cancers are epithelial ovarian cancers (EOCs), which are further classified into different histologic subtypes (5).

For the initial treatment of EOCs, the current practice guidelines recommend extensive cytoreductive surgery followed by platinum-based combination chemotherapy, regardless of histologic subtypes (6). However, there are differences in treatment response and survival outcomes among the histologic subtypes of EOC; therefore, histology is regarded as one of the principal prognostic factors (7). Ovarian clear cell carcinomas (OCCCs), which account for 5–25% of EOCs, are diagnosed at an early stage in younger women (2). According to the literature, OCCC has a relatively worse prognosis, likely because of its higher resistance to platinum-based chemotherapy (8, 9).

OCCC has a distinct carcinogenesis compared to ovarian serous carcinoma (OSC). Previous studies have reported that women with endometriosis, a common benign disease of the female reproductive system, are at an increased risk of developing EOCs, especially OCCC and endometrioid carcinoma (10, 11). Based on molecular pathologic evidence, endometriosis is regarded as a precursor of OCCC (12). Interestingly, the prevalence of OCCC shows geographic variations. OCCC constitutes only a small portion of EOCs in North American and European countries (13, 14), whereas it is more prevalent in East Asian countries; in Japan, OCCC accounts for up to 25% of EOCs (15).

To date, only few studies have reported nationwide survival outcomes of primary OCCC in Korea (3, 16). However, none of these studies directly compared the baseline characteristics and survival of OCCC with those of other histologic subtypes. Although the first-line treatment of OCCC has been relatively locked in a stalemate, that of OSC has been evolving, with poly (adenosine diphosphate–ribose) polymerase (PARP) inhibitor maintenance therapy being introduced to high-grade OSC based on promising results from phase III randomized controlled trials (RCTs) (17, 18). Certain changes in treatment confined to specific histologic subtypes often cause a gap in relative survival within the same primary EOC. Thus, we aimed to investigate trends in the incidence and survival rates of primary OCCC compared to OSC in Korea using a nationwide collected database.

## Materials and Methods

### Patient Selection and Data Collection

In this retrospective analysis, we explored the Korea Central Cancer Registry (KCCR), which covers 98% of the cancer cases in Korea since 1999. Details of the KCCR are described in the previously published study (19). From the KCCR database, we retrieved information of all patients diagnosed with primary EOC between 1999 and 2018 using the topography code C56.9 and morphology codes M8010-8231, M8246-8576, M9110, and M9014-9015 of the International Classification of Diseases for Oncology, 3rd edition (20). We excluded patients diagnosed with non-EOCs, such as sex cord-stromal tumors and germ cell tumors, and mixed EOCs with two different histologic subtypes. Among them, only those with OCCC (M8310-8313, 9110) and OSC (M8441, M8460-8463, M9014) were further identified according to the Cancer Incidence in Five Continents histologic groups (21). Patient information, such as age at diagnosis of EOCs; the Surveillance, Epidemiology, and End Results (SEER) summary stage; and treatment modality, were collected.

### Statistical Analyses

We calculated age-standardized incidence rates (ASRs) by applying Segi’s standard population and annual percent changes (APCs) to estimate trends in the incidence rates for all EOCs, OCCCs, and OSCs. The patients were divided into three groups based on age (<40 years, 40−59 years, and ≥60 years), and age-specific incidence rates were compared.

Differences in characteristics between the OCCC and OSC groups were evaluated. We used Student’s t-test to compare continuous variables and Pearson’s chi-squared test to compare categorical variables. For survival outcomes, we compared the overall survival (OS) between the two groups using Kaplan–Meier analysis with the log-rank test. Subgroup analyses were performed based on the year of diagnosis (1999–2008 and 2009–2018), age at diagnosis (<50 years and ≥50 years), and SEER summary stage (localized, regional, and distant stage).

All statistical analyses were conducted using SAS 9.3 statistical software (SAS Institute Inc., Cary, NC, USA). For statistical tests, a two-tailed *P* value of <0.05 was considered statistically significant.

### Ethical Statements

The Institutional Review Board of the National Cancer Center in Korea approved the study protocol (No. NCC 2021-0346). As the current study was a secondary analysis of de-identified data, the requirement for informed consent was waived.

## Results

Between 1999 and 2018, a total of 2,962 cases of OCCC and 15,320 cases of OSC were included in the analysis. In 2018, of the 2,380 patients diagnosed with EOC, 298 (12.5%) had OCCC and 1,319 (55.4%) had OSC histologic subtypes. During the 20-year period, the ASRs of EOC, OCCC, and OSC were 4.50, 0.44, and 2.23 per 100,000 women, respectively. Overall, the incidence rate of EOC increased gradually, with an APC of 2.74% (*P*<0.0001). Specifically, the incidence rate of OCCC increased markedly (APC, 7.85%; *P*<0.0001) when compared to that of OSC (APC, 3.96%; *P*<0.0001) (**Table 1** and **Supplementary Table 1**).

**Table 1:** The incidence of primary ovarian clear cell carcinoma and serous carcinoma in Korea, 1999−2018.

Year	Clear cell carcinoma		Serous carcinoma	
	ASR per 100,000 women	Cases	ASR per 100,000 women	Cases
1999	0.16	42	1.59	409
2000	0.23	62	1.34	355
2001	0.23	67	1.39	382
2002	0.24	69	1.55	437
2003	0.27	80	1.79	517
2004	0.22	66	1.79	536
2005	0.27	85	1.92	589
2006	0.32	104	1.97	623
2007	0.34	112	2.17	707
2008	0.39	133	2.13	723
2009	0.41	141	2.13	741
2010	0.45	160	2.34	830
2011	0.47	168	2.32	842
2012	0.50	185	2.39	905
2013	0.52	194	2.47	949
2014	0.58	223	2.52	988
2015	0.61	238	2.54	1024
2016	0.65	258	3.01	1255
2017	0.71	277	2.79	1189
2018	0.76	298	2.99	1319
1999−2018	0.44	2962	2.23	15320
APC (%)	7.85		3.96	
*P*-value	<0.0001		<0.0001	

APC, annual percent change; ASR, age-standardized incidence rate.

**Table 2** presents age-specific incidence rates of OCCC and OSC. Between 1999 and 2018, the incidence rates of both histologic subtypes significantly increased across all age groups. The increase in incidence rates was profound in OCCC, with APCs of 8.31% (*P*<0.0001), 7.94% (*P*<0.0001), and 10.49% (*P*<0.0001) for age groups of <40 years, 40−59 years, and ≥60 years, respectively. Baseline and treatment-related characteristics are presented in **Table 3**. Patient age at the time of diagnosis was significantly lower in the OCCC group than in the OSC group (mean, 49.5 vs. 55.4 years, *P*<0.0001). While 50.9% of the patients with OCCC were diagnosed before the age of 50, only 32.0% of patients with OSC were diagnosed before the age of 50. Patients in their 40s constituted the largest proportion (35.3%) in the OCCC group, whereas patients in their 50s constituted the largest proportion in the OSC group.

**Table 2:** Age-specific incidence of primary ovarian clear cell carcinoma and serous carcinoma in Korea.

Characteristics	Clear cell carcinoma	Serous carcinoma
**Age group at diagnosis**		
**<40 years**		
1999	0.05	0.43
2005	0.15	0.35
2012	0.25	0.45
2018	0.35	0.49
APC (%)	8.31	2.80
* P*-value	<0.0001	0.0004
**40-59 years**		
1999	0.61	4.10
2005	0.88	5.18
2012	1.53	6.53
2018	2.53	8.11
APC (%)	7.94	3.93
* P*-value	<0.0001	<0.0001
**≥60 years**		
1999	0.03	4.21
2005	0.17	5.43
2012	0.68	7.06
2018	0.77	9.82
APC (%)	10.49	5.25
* P*-value	<0.0001	<0.0001

APC, annual percent change.

**Table 3:** Characteristics of patients with primary ovarian clear cell carcinoma and serous carcinoma in Korea.

Characteristics	Clear cell carcinoma	Serous carcinoma	*P*-value
Follow-up from registration, years			
Mean (SD)	6.2 (5.1)	5.3 (4.6)	<0.0001
Age at diagnosis, years			
Mean (SD)	49.5 (9.9)	55.4 (11.7)	<0.0001
Case (%)	2962 (100)	15320 (100)	
Age group at diagnosis, years			<0.0001
<30	54 (1.8)	253 (1.7)	
30-39	408 (13.8)	883 (5.8)	
40-49	1046 (35.3)	3766 (24.6)	
50-59	1041 (35.1)	4940 (32.2)	
60-69	310 (10.5)	3514 (22.9)	
70-79	91 (3.1)	1710 (11.2)	
≥80	12 (0.4)	254 (1.7)	
Stage at diagnosis (since 2006)			<0.0001
Local	1251 (50.2)	1407 (11.6)	
Regional	608 (24.4)	2200 (18.2)	
Distant	536 (21.5)	7988 (66.0)	
Unknown	96 (3.9)	500 (4.1)	
Treatment modality			
Surgery			<0.0001
Yes	2863 (96.7)	14125 (92.2)	
No	99 (3.3)	1195 (7.8)	
Radiation therapy			<0.0001
Yes	34 (1.1)	236 (1.5)	
No	2928 (98.9)	15084 (98.5)	
Chemotherapy			<0.0001
Yes	2250 (76.0)	12258 (80.0)	
No	712 (24.0)	3062 (20.0)	

SD, standard deviation.

The SEER summary stage data have been available since 2006 in the KCCR. Primary OCCC was most frequently diagnosed at the localized stage (50.2%), followed by the regional stage (24.4%) and distant stage (21.5%). In contrast, primary OSC was most frequently diagnosed at the distant stage (66.0%), followed by the regional stage (18.2%) and localized stage (11.6%) (**Table 3**).

Regarding the treatment modality within 4 months after first diagnosis, patients with OCCC underwent surgery more frequently than those with OSC (96.7% vs. 92.2%; *P*<0.0001). However, patients with OCCC received less radiation therapy (1.1% vs. 1.5%; *P*<0.0001) and chemotherapy (76.0% vs. 80.0%; *P*<0.0001) during primary treatment than those with OSC (**Table 3**).

**Figure 1:**
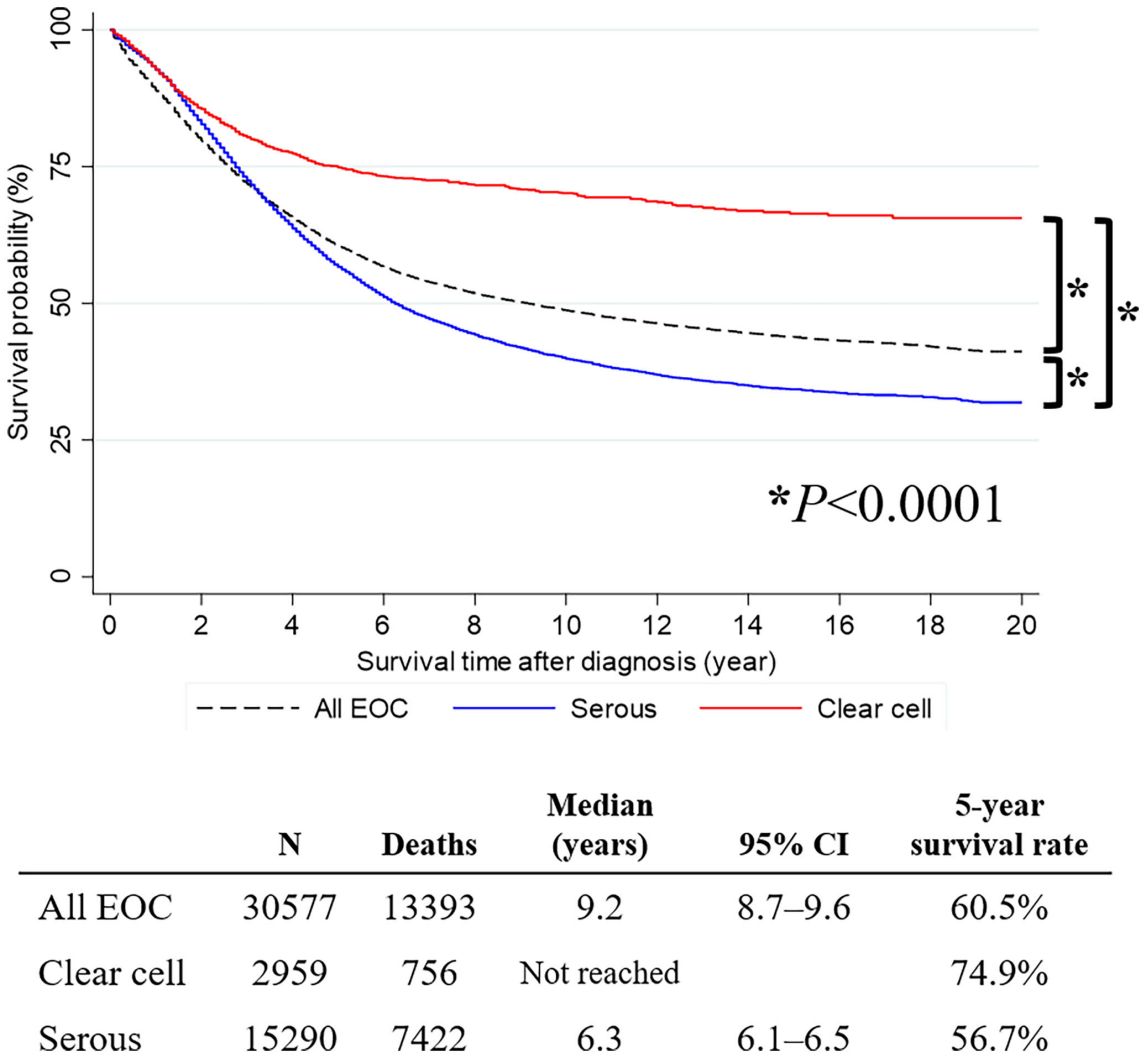
Survival curve of all primary epithelial ovarian cancer, ovarian serous carcinoma, and ovarian clear cell carcinoma cases.

The mean follow-up period of primary OCCC and OSC were 6.2 and 5.3 years from the cancer diagnosis, respectively. Survival analysis revealed that patients with OCCC had significantly better OS than those with OSC (median survival time, not reached vs. 6.3 years; 5-year survival rate, 74.9% vs. 56.7%; *P*<0.0001) (**Figure 1**).

**Figure 2:**
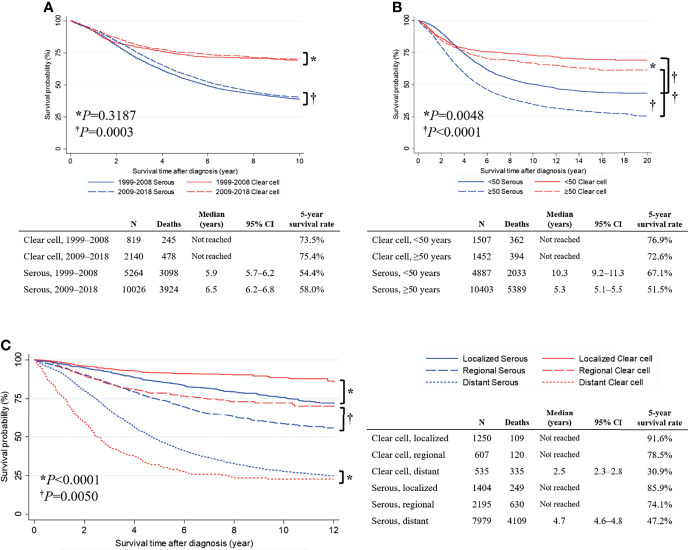
Comparisons of overall survival between primary serous and clear cell carcinoma cases. **(A)** Survival curve by the year of diagnosis; **(B)** Survival curve by the age at diagnosis; **(C)** Survival curve by the SEER summary stage.

Next, we conducted a subgroup analysis based on the year of diagnosis. In the OCCC histologic subtype, no difference in OS was observed between patients diagnosed in 2009–2018 and 1999–2008 (5-year survival rate, 75.4% vs. 73.5%; *P*=0.3187). However, in the OSC histologic subtype, patients diagnosed in 2009−2018 showed significantly better OS than those diagnosed in 1999−2008 (median survival time, 6.5 vs. 5.9 years; 5-year survival rate, 58.0% vs. 54.4%; *P*=0.0003) (**Figure 2A**). We also conducted a subgroup analysis based on the age at diagnosis. In the OCCC histologic subtype, patients diagnosed before the age of 50 years showed significantly improved OS compared to those diagnosed after the age of 50 years (5-year survival rate, 76.9% vs. 72.6%; *P*=0.0048). The same was true for patients with the OSC histologic subtype (median survival time, 10.3 vs. 5.3 years; 5-year survival rate, 67.1% vs. 51.5%; *P <*0.0001) (**Figure 2B**). Applying the SEER summary stage, we observed that the survival of patients with OCCC and OSC significantly differed by stage (both *P* values <0.0001). For the localized stage, patients with OCCC had significantly better OS than those with OSC (5-year survival rate, 91.6% vs. 85.9%; *P*<0.0001). The same was true for the regional stage (5-year survival rate, 78.5% vs. 74.1%; *P*=0.0050). However, an opposite result was observed in the distant stage: patients with OCCC showed a significantly worse OS than those with OSC (median survival time, 2.5 vs. 4.7 years; 5-year survival rate, 30.9% vs. 47.2%; *P*<0.0001) (**Figure 2C**).

## Discussion

From the KCCR database, we identified 2,962 cases of primary OCCC between 1999 and 2018, with an ASR of 0.44 per 100,000 women. Over a period of 20 years, there has been a statistically significant annual increase in OCCC across all age groups in Korea. Early-stage OCCC has a relatively good prognosis. However, advanced-stage OCCC has a worse survival than advanced-stage OSC.

In Korea, the westernization of diet and lifestyle (e.g., increased obesity and excessive estrogen exposure) and changes in reproductive (e.g., earlier menarche) and sociodemographic factors (e.g., decreased childbearing) are collectively responsible for the increase in the incidence of EOCs (3, 4). Notably, we found that the extent of increase was remarkably higher in OCCC than in OSC. Considering changes in the ratio of ASRs (OCCC to OSC) from 0.10 in 1999 to 0.25 in 2018, the incidence of OCCC seems to have increased more than that of OSC.

The prevalence of OCCC varies by geography and ethnicity (13–15). In the current study, OCCC accounted for 4.5% of all EOC cases in 1999 and 12.5% in 2018, which was higher than that observed in the United States (4.8%) (14) but lower than that observed in Japan (25.0%) (15). Similar to our study, an increase in OCCC incidence was observed in a Taiwanese National Cancer Registry study (22). Interestingly, Asian women living in the United States also experienced a significant increase in OCCC between 2000 and 2013 (APC, 1.85%) (23). The marked increase in OCCC in Korean women could be explained as follows. First, it is possible that OCCC, especially in cases with a papillary growth pattern, was often pathologically mistaken for high-grade OSC in the past (24). The current use of immunomarkers might contribute to the improvement in the diagnostic accuracy of OCCC. Second, the high prevalence of endometriosis in Korean women might increase the risk of developing OCCC. In general, endometriosis affects approximately 10% of reproductive-age women, but it has been reported to be more prevalent in Japanese and Korean women than in white women (25). Moreover, according to a Korean national population-based study, there was a significant increase in the diagnosis of endometriosis in young women (<24 years) between 2002 and 2013, with the highest prevalence in women aged 30–34 years (26), which contributed to the recent increase in OCCC incidence.

During the same period, the incidence rate of OSC increased and reached its peak in 2016 (ASR, 3.01 per 100,000 women), after which it did not increase further but remained stagnant for two years. Such a plateau seems to be associated with the recent increase in genetic counseling and *BRCA1/2* gene testing in patients with EOC (especially high-grade OSC) and their families, as well as risk-reducing salpingo-oophorectomy (RRSO) in *BRCA1/2* mutation carriers to protect against EOC (27, 28). The Korean National Health Insurance Service started to cover the *BRCA1/2* gene tests and RRSO in April 2012 and December 2012, respectively. Therefore, we expect the relative incidence rate of OSC to remain unchanged or decrease further.

According to the literature, patients with OCCC are younger and present at an early stage more frequently than those with OSC. According to the SEER database, of the 1,411 patients diagnosed with OCCC between 1988 and 2001, 56.3% had stage I disease and 11.0% had stage II disease (14). In a Japanese multicenter retrospective cohort study of 101 patients diagnosed with OCCC between 1988 and 1998, 48.5% had stage I disease and 9.9% had stage II disease (15). Similar findings were observed in the present study: 50.2% of patients with OCCC were diagnosed with localized disease, whereas only 11.6% of patients with OSC were diagnosed at the localized stage (*P <*0.0001). Such differences in the initial stage distribution between OCCC and OSC may be attributed to the tendency of OCCCs to have larger tumor sizes than OSCs, which is a clue for differentiation of EOC histologic subtypes in imaging studies (29). In addition, because of its association with endometriosis, OCCC tends to be diagnosed earlier, including cases with incidental OCCC during endometriosis surgery and cases with early detection of malignant transformation during endometriosis surveillance (30).

Whether the coexistence of endometriosis affects the prognosis of OCCC remains controversial. While some studies reported that endometriosis in OCCC was associated with better survival outcomes due to favorable manifestations such as early-stage disease (31, 32), other studies concluded that endometriosis may not affect survival outcomes (33, 34). Nevertheless, the innate limitations of KCCR hindered us from differentiating between patients with OCCC with endometriosis and those without endometriosis. We were also unable to investigate the effect of endometriosis on the survival outcomes.

Notably, we observed that patients with OCCC with distant-stage disease had a significantly worse OS than those with OSC (*P*<0.0001). Similarly, according to a previous meta-analysis, patients with stage III–IV OCCC (n=845) had a higher risk of mortality than those with stage III–IV OSC (n=18,471; pooled hazard ratio, 1.71; 95% CI, 1.57–1.86) (9). Similar results were observed in a Taiwanese single-center study (35). One possible underlying reason for the relatively poor prognosis in advanced-stage OCCC is a lower response rate to first-line, second-line, and subsequent lines of chemotherapy when compared to OSC (36). Interestingly, while there was no improvement in OS between patients diagnosed with OCCC in 2009−2018 and 1999−2008, a significant improvement in OS was observed in patients diagnosed with OSC in 2009−2018 when compared to those diagnosed in 2009−2018. Based on these findings, we inferred that there has been relatively little improvement with therapeutic agents in OCCC than in OSC. Considering that the Korean Ministry of Food and Drug Safety recently approved maintenance therapy with PARP inhibitors in selected patients with primary and platinum-sensitive relapsed, high-grade OSC, we expect further improvement in survival outcomes in high-grade OSC.

Our study results suggest that there is room for improvement in the management of patients with OCCC, especially those with distant stages, and the development of early detection methods and novel therapeutic agents is further emphasized. Previous molecular genetic studies revealed distinct features of OCCC compared to high-grade OSC. OCCC is known to have few *TP53* mutations but frequent *ARID1A* and *PIK3CA* mutations; it also activates the PI3K/AKT/mTOR pathway (37). Therefore, targeting the PI3K/AKT/mTOR signaling pathway has been suggested as a therapeutic target in OCCC. For example, in a phase II clinical trial, temsirolimus, an mTOR inhibitor, was combined with carboplatin and paclitaxel, followed by temsirolimus consolidation as first-line therapy in stage III–IV OCCC. However, this regimen failed to prolong the progression-free survival (38). Owing to its rarity, the number of previous and ongoing clinical trials on OCCC is relatively less than that of OSC. Nevertheless, efforts have been made to devise specific treatments for OCCC. For example, a phase II clinical trial reported minimal efficacy of sunitinib monotherapy in recurrent OCCC patients (39). Currently, a phase II RCT, named MOCCA, is ongoing, which investigates the efficacy of durvalumab, an immune checkpoint inhibitor, compared to standard chemotherapy in patients with recurrent OCCC (40). Like these trials, further studies exploring optimal treatment strategies for OCCC are encouraged.

Our study has several limitations. First, we could not obtain the following information from the KCCR database: patients’ demographic characteristics other than age at diagnosis, performance status, initial serum CA-125 levels, International Federation of Gynecology and Obstetrics stage, and detailed information on treatments. For example, it is hard to know how many patients underwent primary cytoreductive surgery or neoadjuvant chemotherapy followed by interval cytoreductive surgery, and achieved complete resection after surgery. Although anatomical sites of primary disease and residual tumors might be associated with recurrence patterns (41), such information was unavailable at this moment. Information on chemotherapy regimens was also unavailable. In particular, bevacizumab, a humanized anti-vascular endothelial growth factor monoclonal antibody, was incorporated in the primary treatment of EOC in February 2013 after approval by the Korean Ministry of Food and Drug Safety. However, we could not identify bevacizumab users in the study population. As these factors might influence the prognosis of patients with OCCC and OSC, a novel, national-level strategy for data acquisition is needed. Second, during the study period, we could not determine how the changes in pathologic criteria influenced the incidence of OCCC and OSC. In particular, a two-tier classification of OSC, high- and low-grade tumors, has not yet been adopted. Lastly, among the various parameters of survival outcomes, only OS was presented. Other parameters, such as progression-free survival and quality-adjusted survival, could not be calculated from the database.

Despite these limitations, we could clearly demonstrate serial trends in incidence rates and survival of OCCC compared to those of OSC over a period of 20 years in Korea using the KCCR database, which covers almost all patients with primary EOC. Clinical unmet needs surrounding OCCC were also clearly identified. We expect that the recent revision of the Cancer Control Act in Korea will allow researchers to combine the KCCR database, National Health Insurance Corporation database, Health Insurance Review & Assessment Service database, and the Statistics Korea database. It will be possible to conduct more precise, big-data-driven, integrative cancer research in the near future.

In conclusion, our study using the KCCR database demonstrated that the incidence of OCCC significantly increased from 1999 to 2018 in Korea. Patients with OCCC were significantly younger and had early-stage disease more frequently than those with OSC. Early-stage OCCC had a relatively good prognosis, but advanced-stage OCCC had a worse survival than advanced-stage OSC. Further studies investigating the development of early detection methods and novel therapeutic agents are warranted to improve the survival outcomes of patients with OCCC.

## Data Availability Statement

The raw data supporting the conclusions of this article will be made available by the authors, without undue reservation.

## Ethics Statement

The Institutional Review Board of the National Cancer Center in Korea approved the study protocol (No. NCC 2021-0346). As the current study was a secondary analysis of de-identified data, the requirement for informed consent was waived. Written informed consent for participation was not required for this study in accordance with the national legislation and the institutional requirements.

## Author Contributions

SIK: designed the study and wrote the manuscript. HIH and KJE: interpreted data and reviewed the manuscript. JL: contributed to analysis of the data and wrote the manuscript. Y-JW and MCL: designed the study, acquired and interpreted data, reviewed the manuscript, and approved the final report.

## Funding

This work was supported by the National Cancer Center of Korea (Grant No. 1910132).

## Conflict of Interest

The authors declare that the research was conducted in the absence of any commercial or financial relationships that could be construed as a potential conflict of interest.

## Publisher’s Note

All claims expressed in this article are solely those of the authors and do not necessarily represent those of their affiliated organizations, or those of the publisher, the editors and the reviewers. Any product that may be evaluated in this article, or claim that may be made by its manufacturer, is not guaranteed or endorsed by the publisher.
