# Supplementary material for: Trends in the Incidence and Survival Rates of Primary Ovarian Clear Cell Carcinoma Compared to Ovarian Serous Carcinoma in Korea
Source: Front Oncol. 2022 Apr 7;12:874037. doi: 10.3389/fonc.2022.874037 (PMC9021727; doi:10.3389/fonc.2022.874037)
Supplement: Supplementary file 1 [file Table_1.docx]

**Supplementary Table 1.** The incidence of primary epithelial ovarian cancer in Korea, 1999−2018

| **Year** | **Epithelial ovarian cancer** | |
| --- | --- | --- |
|  | **ASR**  **per 100,000 women** | **Cases** |
| 1999 | 3.55 | 931 |
| 2000 | 3.51 | 952 |
| 2001 | 3.46 | 961 |
| 2002 | 3.56 | 1018 |
| 2003 | 3.78 | 1095 |
| 2004 | 3.78 | 1137 |
| 2005 | 3.94 | 1216 |
| 2006 | 4.04 | 1286 |
| 2007 | 4.46 | 1455 |
| 2008 | 4.23 | 1433 |
| 2009 | 4.15 | 1450 |
| 2010 | 4.58 | 1602 |
| 2011 | 4.54 | 1634 |
| 2012 | 4.85 | 1798 |
| 2013 | 4.77 | 1822 |
| 2014 | 5.17 | 1988 |
| 2015 | 5.05 | 1995 |
| 2016 | 5.54 | 2236 |
| 2017 | 5.52 | 2250 |
| 2018 | 5.70 | 2380 |
| 1999−2018 | 4.50 | 30639 |
| APC (%) | 2.74 | |
| *P*-value | <0.0001 | |
| Abbreviations. APC, annual percent change; ASR, age-standardized incidence rate. | | |
